# Phytochemical and Cytotoxic Aspects of Amaryllidaceae Alkaloids in *Galanthus* Species: A Review

**DOI:** 10.3390/plants13243577

**Published:** 2024-12-21

**Authors:** Borislav Georgiev, Boriana Sidjimova, Strahil Berkov

**Affiliations:** Institute of Biodiversity and Ecosystem Research, Bulgarian Academy of Sciences, 1113 Sofia, Bulgaria; sidjimova@yahoo.com

**Keywords:** *Galanthus*, Amaryllidaceae alkaloids, biological activities, cytotoxicity, anticancer

## Abstract

The genus *Galanthus* (Amaryllidaceae) currently contains 25 plant species naturally occurring in Europe and the Middle East region. These perennial bulbous plants possess well-known medicinal and ornamental qualities. Alkaloid diversity is their most distinctive phytochemical feature. A total of 127 compounds (≈20% of all known Amaryllidaceae alkaloids) grouped in 16 structural types have been previously found in *Galanthus* extracts. Some structural types like galanthindole, graciline and plicamine were first discovered in *Galanthus* plants. Nine *Galanthus* species, however, remain unstudied regarding their alkaloid patterns. Intraspecific variability has only been studied in *G. nivalis* and *G. elwesii*. Amaryllidaceae alkaloids are molecules with anticholinesterase, antibacterial, antifungal, antiviral and anticancer properties. Galanthamine, isolated for the first time from *Galanthus woronowii* Losinsk., stands out as an acetylcholinesterase inhibitor approved for medical use by the FDA for the treatment of symptoms of Alzheimer’s disease. Lycorine, narciclasine and pancratistatin are noteworthy cytotoxic and antitumor alkaloids. Structural types like galanthamine, homolycorine and haemanthamine are fairly well studied in anticancer research, but little to no information is available on galanthindole, graciline and other types. This review aims to present an update on the alkaloid diversity of *Galanthus* spp. and highlight the need for further research on the antitumor potential of these molecules.

## 1. Introduction

The Amaryllidaceae family contains three subfamilies, 71 genera and more than 1600 species. The genus *Galanthus* (snowdrops, from the Greek gala, which means ‘milk’, and ánthos, which means ‘flower’) comprises 25 species placed in the Amaryllidoideae subfamily of the Amaryllidaceae family [1]. Snowdrops are bulbous perennial herbaceous plants with two leaves and bell-shaped white flowers with green spots. The flowers contain two whorls of three free perianth segments each, with the inner three being smaller than the outer three.

*Galanthus* species grow naturally in Europe and the Middle East region (with western, eastern, and southern limits being the Pyrenees (France, Spain); Caucasus (Iran); and Sicily, Greece, Turkey, Lebanon and Syria, respectively). Due to introduction and cultivation in other countries, the northern limit of natural distribution is unknown [2]. Wild snowdrops can be typically found in cool places with plenty of water and damp base-rich soil or soil overlying basic rocks (e.g., limestone). They reside in the shades of deciduous or mixed woodlands, woodland edges and grassland, amongst large rocks, near water sources like rivers or streams and often in mountainous regions (800–1500 m). Woodlands often include the tree genera *Fagus*, *Alnus*, *Carpinus*, *Quercus*, *Abies*, *Pinus*, *Taxus*, etc. Most but not all snowdrops have a winter or early spring flowering, meaning they can withstand temperatures even below 0 °C [3].

Some *Galanthus* species have been included in the IUCN Red List of Threatened Species, and their status is updated periodically [4]. Their collection is prohibited or restricted in most countries.

The identification of species is particularly difficult considering the morphological similarities within the genus and the lack of unambiguous morphological characteristics. The current taxonomy of the genus is based on molecular phylogenetics studies of the internal transcribed spacer (ITS) and plastid DNA [2]. A list of accepted *Galanthus* species and their geographical distributions are shown in Table 1.

Snowdrops are world famous ornamental and garden plants with high economic value. For example, Turkey’s 2024 export quota for *G. elwesii* bulbs is set at 7.5 million (5.5 million wild and 2 million artificially propagated) [5]. The oldest recorded use of *Galanthus* spp. in traditional medicine might be the one described by Homer, who defined ‘moly’ as an antidote for poisoning. Currently, snowdrops are not particularly common in traditional medicine but have been used to treat pain, migraines and headaches. The herbs are reported to have cardiotonic, stomachic and emmenagogue properties, whereas the poultice prepared from the fresh underground parts has external use in abscess maturation [6,7]. Furthermore, in Bulgaria and Georgia, bulb decoctions from *G. nivalis* and *G. woronowii*, respectively, were used for poliomyelitis, and the former was used for colds and fevers as well [8]. Snowdrops are a rich source of secondary metabolites, such as flavonoids, phenolics, terpenoids and alkaloids, with a broad spectrum of biological and pharmacological activities. The well-established alkaloid diversity among *Galanthus* spp. and populations serves as a base for the discovery of anticholinesterase, antibacterial, antifungal, antimalarial, antiviral, antioxidant, anticancer and anti-inflammatory properties.

## 2. Structural Types and Biosynthetic Pathways of Amaryllidaceae Alkaloids

**Figure 1:**
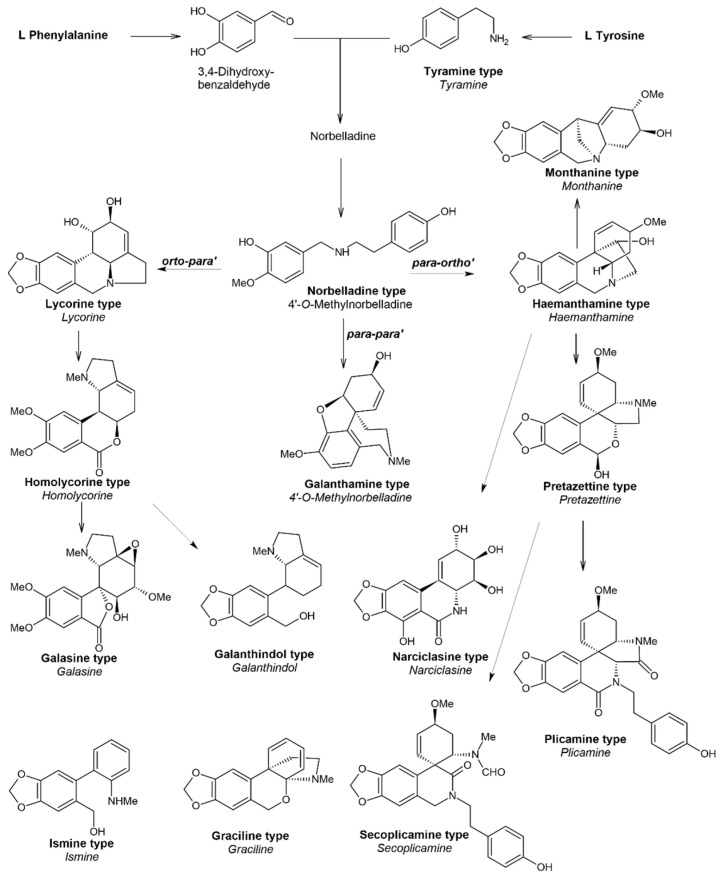
Biosynthetic pathways of structural types of Amaryllidaceae alkaloids found in *Galanthus* spp. (adapted from Berkov et al., 2020 [9]).

Amaryllidaceae alkaloids are the most important phytochemical feature of all plants in the Amaryllidoideae subfamily. They are nitrogen-containing secondary metabolites grouped mainly in 18 structural types, namely norbelladine, lycorine, homolycorine, galasine, galanthindole, crinine, haemanthamine, cripowellin, narciclasine, pretazettine, plicamine, secoplicamine, graciline, montanine, galanthamine, ismine, *Sceletium* and miscellaneous (Figure 1). Currently, more than 630 alkaloids are known, and new structures continue to be discovered [9].

Three main groups of enzymes are involved in the process of generating Amaryllidaceae alkaloids: oxidoreductases, transferases and lyases. Based on the action of these enzymes, many alkaloids are intermediates instead of endpoints; for example, galanthine is produced after the *O*-methylation of methylpseudolycorine, which forms after the hydroxylation of pluviine [10].

The biosynthesis of Amaryllidaceae alkaloids starts with an intramolecular oxidative coupling of two amino acid (L-tyrosine and L-phenylalanine) derivatives resulting in the formation of norbelladine. Then, a key enzymatic reaction takes place where norbelladine is methylated by norbelladine 4′-*O*-methyltransferase to form 4′-*O*-methylnorbelladine. Research on *O*-methyltransferases from different plant origins led to the conclusion that *para*-*O*-methylation is preferred over *meta*- due to the presence of an electron-donating amine group in the substrate (as in norbelladine) [10].

4′-*O*-Methylnorbelladine is an intermediate metabolite that can undergo three patterns of C–C phenol oxidative coupling. After coupling, alkaloid precursors are formed, which participate in further structural rearrangements to produce all structural types (Figure 1) [9,10]. The three pathways of C–C coupling of 4′-*O*-methylnorbelladine are *para*–*ortho’*, *ortho*–*para’* and *para*–*para’*. *Para*–*ortho’* coupling produces the galanthamine type through nornarwedine. *Ortho*–*para’* coupling results in noroxopluviine, whose routes of modification lead to lycorine and homolycorine types. *Para*–*para’* coupling results in the formation of noroxomaritidine with two enantiomers: 10b*S*,4a*R*-, a precursor for 5,10b-ethanophenanthridine (crinine and haemanthamine) and pretazettine types, and 10b*R*,4a*S*-, a precursor for narciclasine and montanine types. The ismine type is generated from noroxomaritidine as well as by a catabolic pathway for which only limited information is available [10]. C–C phenol coupling reactions are primarily catalyzed by cytochrome P450 (CYP) enzymes. CYP96 is a notable enzyme group involved in the modification of 4′-*O*-methylnorbelladine in Amaryllidaceae plants. Currently, three different types of CYP96T enzymes are suggested to be involved in C–C phenol coupling, namely CYP96T6, CYP96T5 and CYP96T1, catalyzing *para*–*ortho’*, *ortho*–*para’* and *para*–*para’* couplings, respectively [11]. Although a lot is still unknown about the enzymes and corresponding genes that ensure the biosynthesis of Amaryllidaceae alkaloids, the advancement of omics enables the possibility of coexpression and transcriptome analyses.

## 3. Alkaloid Constituents of *Galanthus* spp.

The search for articles on this topic was based on several criteria: *Galanthus* or snowdrop mentioned in the title and/or as a keyword; a time frame of 1995–May 2024; the use of the NCBI PubMed, Google Scholar and ResearchGate databases; TLC/GC-MS/NMR data, preferably in the Methods or Results Section; and an assessment of other secondary metabolites omitted (terpenoids, flavonoids, phenolics, etc.).

Amaryllidaceae alkaloids in snowdrops were first reported in the 1950s, when galanthine [12] and galanthamine [13] were isolated from *G. woronowii* Losinsk. To the best of our knowledge, to date, 16 out of 25 *Galanthus* species have been phytochemically studied (Table 2). According to the number of articles featuring an alkaloid analysis, *G. elwesii* is the most studied, and *G. alpinus*, *G. peshmenii*, *G. platyphyllus*, *G. reginae-olgae*, *G. trojanus* and *G.* × *valentinei* are tied for the least studied. For nine *Galanthus* species, namely *G. panjutinii*, *G. lagodechianus*, *G.* × *allenii*, *G. angustifolius*, *G. koenenianus*, *G. transcaucasicus*, *G. samothracicus*, *G. bursanus* and *G. lebedevae*, no information about their alkaloid contents is available yet. The numbers of articles found from different countries are as follows: Turkey (26), Bulgaria (9), Georgia (2), Italy (1) and Czech Republic (1).

**Figure 2:**
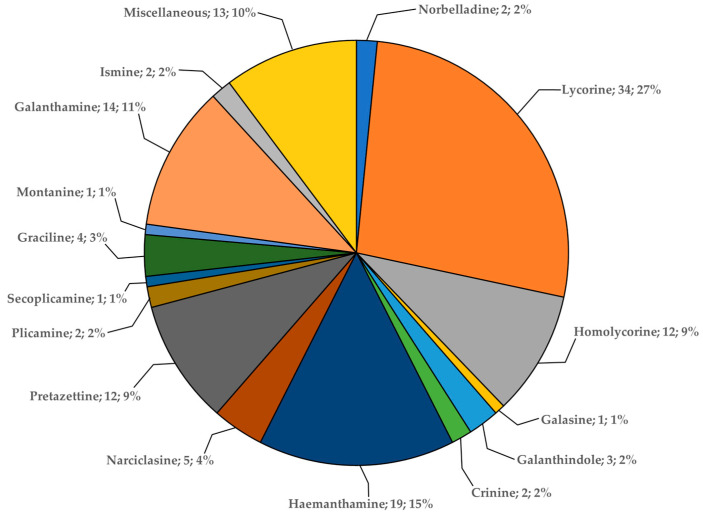
Distribution of structural types of Amaryllidaceae alkaloids reported in *Galanthus* spp.

**Figure 3:**
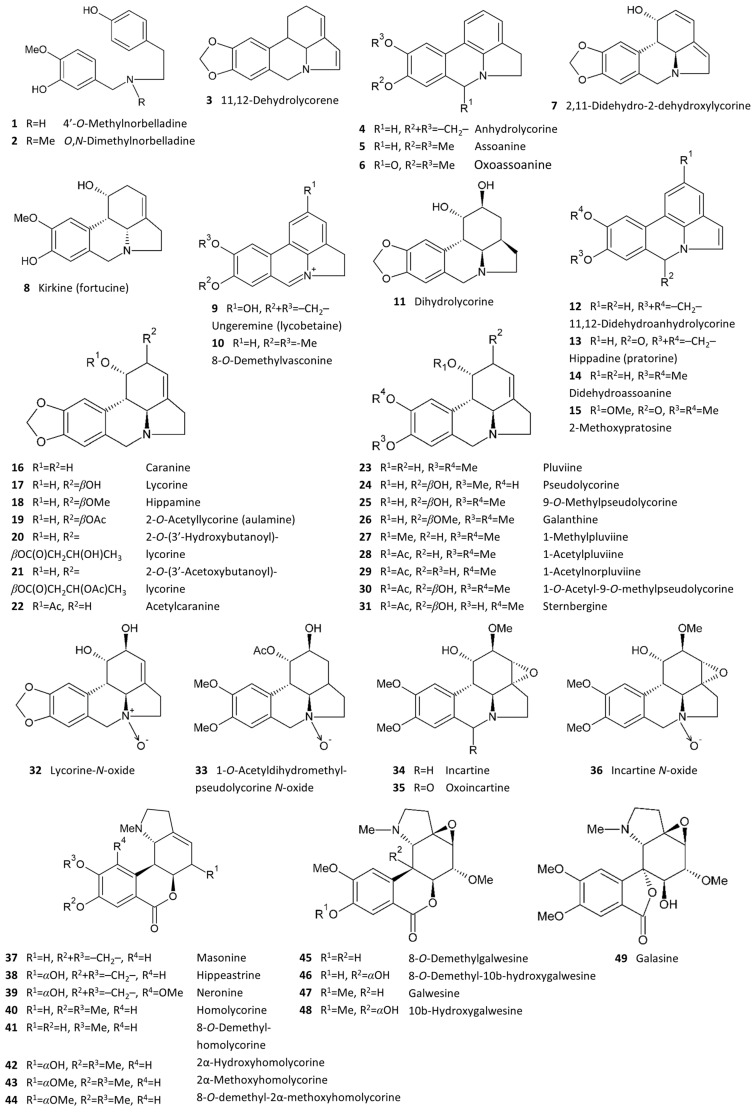

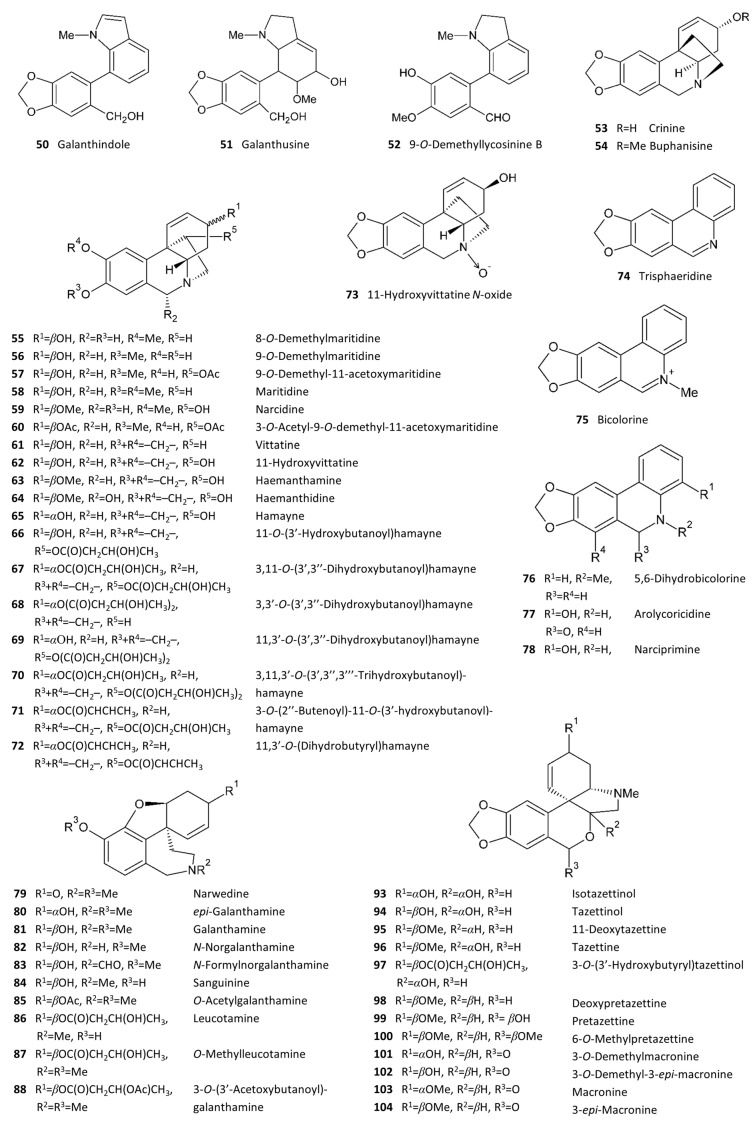

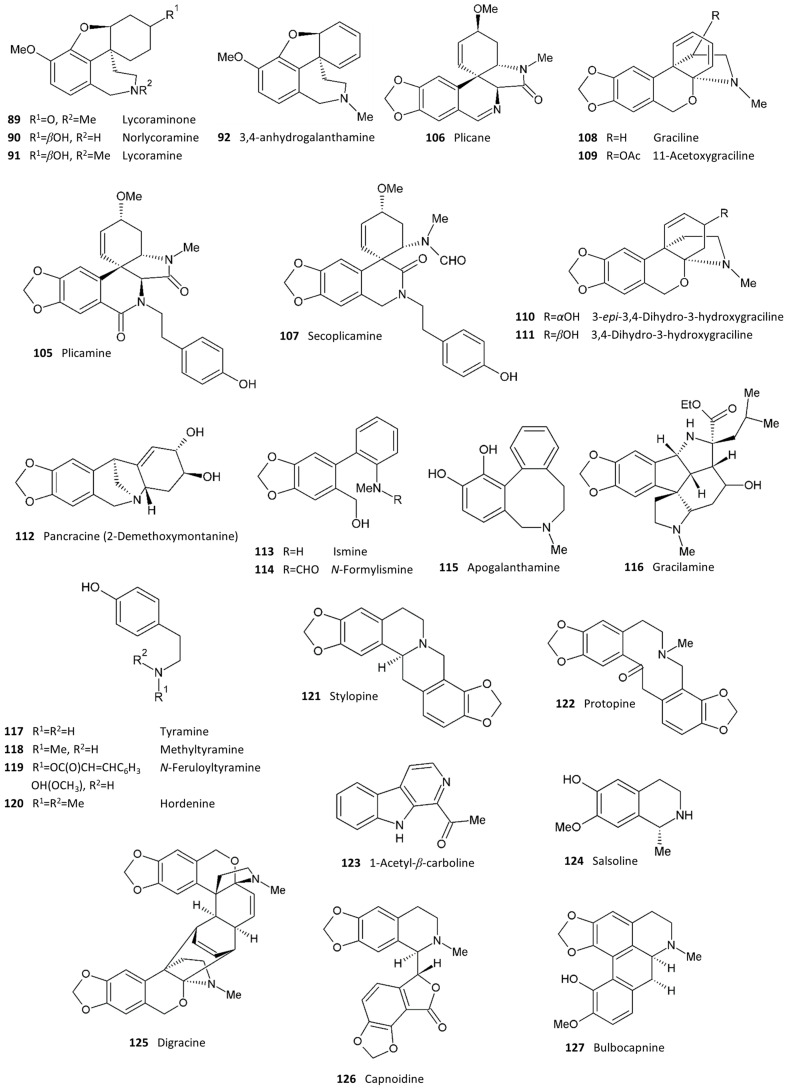
Structures of all Amaryllidaceae alkaloids reported in *Galanthus* spp.

Among the 16 studied *Galanthus* species, a total of 127 alkaloids of 16 structural types have been reported (Table 2; Figure 2 and Figure 3). In this review, the naming of compounds follows Berkov et al., 2020 [9]. The Cripowellin and *Sceletium* types are the only two that have not yet been reported in snowdrops. *Galanthus* spp. were the first known source of some structural types, for example, galanthindole, graciline and plicamine [14,15]. The lycorine type is the most abundant among all known alkaloids [9], so, unsurprisingly, it is the most commonly occurring type in snowdrops as well, with 34 compounds (Figure 2). The number of miscellaneous alkaloids is significant (12), reported in 11 of the 16 studied species. The largest number of alkaloids, significantly more than in any other species, was described in *G. elwesii*–75, followed by *G. nivalis* (35), *G. rizehensis* (27) and *G. gracilis* (26). The lowest numbers of alkaloids were found in *G. platyphyllus* (3), *G. reginae-olgae* (4), *G. ikariae* (7) and *G. peshmenii* (7). The remaining species have about 20 described alkaloids each.

Several articles describe intraspecific differences in the alkaloid patterns of Bulgarian or Turkish populations of *G. elwesii* [16,17,18,19], a species with a very wide geographical distribution. The distribution of the chemotypes indicates that adjacent populations often possess relatively similar alkaloid profiles, and populations living in regions distant from each other synthesize different alkaloids.

The most abundant structural types in separate populations are lycorine, homolycorine, haemanthamine, galanthamine and tyramine. The tyramine type is primarily found in *G. elwesii* and in much lower levels in *G. nivalis*. Since the last review on *Galanthus* alkaloids [20], 16 compounds were reported in *G. elwesii* only, namely oxoassoanine (**6**), 2,11-didehydro-2-dehydroxylycorine (**7**), kirkine (fortucine) (**8**), didehydroassoanine (**14**), 2-methoxypratosine (**15**), 1-methylplyviine (**27**), 1-acetylnorpluviine (**29**), 2α-methoxyhomolycorine (**43**), 9-*O*-demethyllycosinine B (**52**), haemanthidine (**64**), 11,3′-*O*-(dihydrobutyryl)hamayne (**72**), bicolorine (**75**), lycoraminone (**89**), *N*-norlycoramine (*N*-demethyllycoramine) (**90**), 3,4-anhydrogalanthamine (**92**) and apogalanthamine (**115**).

Two articles describe the intraspecific differences in the alkaloid contents of Bulgarian *G. nivalis* populations [17,19] based on GC–MS analyses of their alkaloid fractions. Here, the lycorine, haemanthamine, narciclasine, pretazettine and galanthamine types are predominant, but a large percentage of unknown compounds is present due to the lack of reference MS spectra, possibly hinting at new structures and enabling the opportunity for further research. Since the last review on *Galanthus* alkaloids [20], one compound was reported in *G. nivalis* only: tazettinol (3-*O*-demethyltazettine) (**94**).

*G. nivalis* and *G.* × *valentinei* are the only species rich in hamayne and its hydroxybutanoyl derivatives. *G. nivalis* is one of the two species that make the × *valentinei* hybrid, so such similarities are expected. Furthermore, in *G. nivalis*, such derivatives have also been discovered for lycorine [21,22,23,24].

Galanthamine-type alkaloids are major constituents of some *G. elwesii* populations and are not that common in *G. nivalis*. However, unlike *G. elwesii*, it is noted that in *G. nivalis*, the galanthamine type is represented mainly by lycoramine and its derivatives [17].

Crinine and buphanisine, with a 5,10b-ethano bridge in the β-position, have been reported on several occasions in *Galanthus* spp. after GC–MS analyses. Assignment of the 5,10b-ethano bridge configuration is only possible after isolation and CD analysis [9].

**Table 2:** The distribution of Amaryllidaceae alkaloids in *Galanthus* spp. reported in data in the literature.

	*G. alpinus*	*G. cilicicus*	*G. elwesii*	*G. fosteri*	*G. gracilis*	*G. ikariae*	*G. krasnovii*	*G. nivalis*	*G. peshmenii*	*G. platyphyllus*	*G. plicatus*	*G. reginae-olgae*	*G. rizehensis*	*G. trojanus*	*G. woronowii*	*G.* × *valentinei*
**Norbelladine type**																
4′-*O*-methylnorbelladine (**1**)	[25]				[24]		[24]						[26]	[27]		
*O*,*N*-dimethylnorbelladine (**2**)				[28,29]												
**Lycorine type**																
11,12-dehydrolycorene (**3**)			[18]		[24]		[24]									
anhydrolycorine (**4**)			[17,18,19,20,21,22,23,24,25,26,27,28,29]	[29]			[25]	[17,22]					[30]		[31]	
assoanine (**5**)		[32]	[18]	[29]									[30]		[33]	[23]
oxoassoanine (**6**)			[19]													
2,11-didehydro-2-dehydroxylycorine (**7**)			[18]													
kirkine (fortucine) (**8**)			[19]													
ungeremine (lycobetaine) (**9**)								[21]								
8-*O*-demethylvasconine (**10**)			[34]													
dihydrolycorine (**11**)														[27]		
11,12-didehydroanhydrolycorine (**12**)			[17,18,19]	[29]	[35]		[24]	[17,19]					[30]			[23]
hippadine (pratorine) (**13**)			[19]					[19]								
didehydroassoanine (**14**)			[19]													
2-methoxypratosine (**15**)			[19]													
caranine (**16**)								[22]								
lycorine (**17**)	[25,36,37]	[38]	[16,17,18,22,34,39,40,41,42]	[29]	[24,35,43]	[40,44,45]	[36]	[17,21,22,34,42]		[36]	[43]		[26,30]		[31,33,36]	[23]
hippamine (**18**)			[18]										[30]			
2-*O*-acetyllycorine (aulamine) (**19**)															[31]	
2-*O*-(3′-hydroxybutanoyl)lycorine (**20**)			[17,34]					[17,22]							[31,33]	
2-*O*-(3′-acetoxybutanoyl)lycorine (**21**)			[34]					[17,21,22]							[31]	
acetylcaranine (**22**)													[30]			
pluviine (**23**)					[35]											
pseudolycorine (**24**)							[24]									
9-*O*-methylpseudolycorine (**25**)				[28,29]											[31]	
galanthine (**26**)	[36,37]	[32]	[17,18,19,39]	[28,29]				[17]					[30]		[31,33,36]	
1-methylplyviine (**27**)			[19]													
1-acetylpluviine (**28**)			[19]										[30]			
1-acetylnorpluviine (**29**)			[19]													
1-*O*-acetyl-9-*O*-methylpseudolycorine (**30**)															[31,33]	
sternbergine (**31**)													[30]		[31,33]	
lycorine *N*-oxide (**32**)													[26]			
1-*O*-acetyldihydromethylpseudolycorine *N*-oxide (**33**)														[27]		
incartine (**34**)		[32]	[17,18,22,34,41,42]	[28,29]				[17,22,42]					[26,30]			[23]
oxoincartine (**35**)			[18]	[28,29]												
incartine *N*-oxide (**36**)													[26]			
**Homolycorine type**																
masonine (**37**)			[19,22]													
hippeastrine (**38**)			[17,18,19,22,41,42]		[24]											
neronine (**39**)												[46]				
homolycorine (**40**)	[25]		[17,19,22]		[24,35]				[24]							
8-*O*-demethylhomolycorine (**41**)	[25,36,37]		[17,18,19,22,39,40,41]		[24,35]	[40]			[24]						[36]	
2α-hydroxyhomolycorine (**42**)			[17]													
2α-methoxyhomolycorine (**43**)			[19,22]													
8-*O*-demethyl-2α-methoxyhomolycorine (**44**)			[17,19,22,39]													
8-*O*-demethylgalwesine (**45**)			[17,19,22,39,42]													
8-*O*-demethyl-10b-hydroxygalwesine (**46**)			[39]													
galwesine (**47**)			[16,17,18,19,22,39,42]	[29]				[19]	[24]							
10b-hydroxygalwesine (**48**)			[39]													
**Galasine type**																
galasine (**49**)			[18,39]													
**Galanthindole type**																
galanthindole (**50**)		[32]	[18]	[29]	[24,35]			[19]	[24]		[14]					[23]
galanthusine (**51**)	[36,37]														[36]	
9-*O*-demethyllycosinine B (**52**)			[19]													
**Crinine type**																
crinine (**53**)				[29]		[44,45]						[46]				
buphanisine (**54**)			[16,17,40,42]													
**Haemanthamine type**																
8-*O*-demethylmaritidine (**55**)			[18]		[35]		[24]						[30]	[27]		[23]
9-*O*-demethylmaritidine (**56**)				[29]												
9-*O*-demethyl-11-acetoxymaritidine (**57**)				[28,29]												
maritidine (**58**)			[16,17]													
narcidine (**59**)														[27]		
3-*O*-acetyl-9-*O*-demethyl-11-acetoxymaritidine (**60**)				[28,29]												
vittatine (**61**)		[38]	[17,22,42]	[28]	[24]		[24]						[26,30]			[23]
11-hydroxyvittatine (**62**)		[38]	[18,39]	[29]	[24]		[24]	[42]			[14]		[26,30]			
haemanthamine (**63**)		[32]	[16,17,18,22,40,42,47,48]					[17]						[27]		[23]
haemanthidine (**64**)			[42]													
hamayne (**65**)		[32]	[34,40]			[40,44,45]		[21,22,34]								[23]
11-*O*-(3′-hydroxybutanoyl)hamayne (**66**)			[17,34]					[17,21,22,34,42]								[23]
3,11*-O*-(3′,3″-dihydroxybutanoyl)hamayne (**67**)								[17,21,22,34]								[23]
3,3′-*O*-(3′,3″-dihydroxybutanoyl)hamayne (**68**)								[34]								
11,3′-*O*-(3′,3″-dihydroxybutanoyl)hamayne (**69**)								[34]								
3,11,3′-*O*-(3′,3″,3′′′-trihydroxybutanoyl)hamayne (**70**)								[21,34]								
3-*O*-(2″-butenoyl)-11-*O*-(3′-hydroxybutanoyl)hamayne (**71**)								[17,21,22]								[23]
11,3′-*O*-(dihydrobutyryl)hamayne (**72**)			[19]													
11-hydroxyvittatine *N*-oxide (**73**)														[27]		
**Narciclasine type**																
trisphaeridine (**74**)		[32]	[16,17,18,19,22,42]	[29]	[35]		[24]	[17,19,22,42]			[49]		[30]			[23]
bicolorine (**75**)			[19]													
5,6-dihydrobicolorine (**76**)		[32]	[18]	[29]	[24,35]		[24]									[23]
arolycoricidine (**77**)	[25]												[50]	[27]		
narciprimine (**78**)													[50]			
**Galanthamine type**																
narwedine (**79**)		[32]	[16,17,18,19,39]												[31,33]	
3-*epi*-galanthamine (**80**)			[17]													
galanthamine (**81**)	[36,37]	[32]	[16,17,18,19,39,40,47,48]	[28,29]		[40,44,45]	[36]	[17,19]		[36]		[46]	[30]		[31,33,36]	[23]
*N*-norgalanthamine (*N*-demethylgalanthamine) (**82**)			[16,17,19,39]												[31]	
*N*-formylnorgalanthamine (**83**)			[16,17,19]													
sanguinine (**84**)		[32]	[18,39]												[31,33]	
*O*-acetylgalanthamine (**85**)															[31]	
leucotamine (**86**)			[17,39]													
*O*-methylleucotamine (**87**)			[16,18,19,39]												[31,33]	
3-*O*-(3′-acetoxybutanoyl)galanthamine (**88**)															[34]	
lycoraminone (**89**)			[19]													
*N*-norlycoramine (*N*-demethyllycoramine) (**90**)			[16]													
lycoramine (**91**)			[17,19]					[17]								
3,4-anhydrogalanthamine (**92**)			[17,19]													
**Pretazettine type**																
isotazettinol (**93**)					[51]											
tazettinol (3-*O*-demethyltazettine) (**94**)								[19]								
11-deoxytazettine (**95**)			[17]		[35]			[17,22]					[30]		[31]	
tazettine (**96**)	[36,37]	[32]	[16,17,18,40,42,48]		[24,35]	[40,44]	[36]	[17,21,22,34,42]	[24]	[36]		[46]	[30]		[36]	[23]
3-*O*-(3′-hydroxybutyryl)tazettinol (**97**)											[49]					
deoxypretazettine (**98**)																[23]
pretazettine (**99**)			[19]					[19]								
6-*O*-methylpretazettine (**100**)		[32]	[17,18]		[24]			[17]								[23]
3-*O*-demethylmacronine (**101**)					[24,51]											
3-*O*-demethyl-3-*epi*-macronine (**102**)											[51]					
macronine (**103**)			[19]					[17,19,22]								[23]
3-*epi*-macronine (**104**)		[32]	[18]					[17,19,22,34,42]					[30]			[23]
**Plicamine type**																
plicamine (**105**)											[52]					
plicane (**106**)											[49]					
**Secoplicamine type**																
secoplicamine (**107**)											[52]					
**Graciline type**																
graciline (**108**)					[53]				[24]							
11-acetoxygraciline (**109**)					[53]											
3-*epi*-3,4-dihydro-3-hydroxygraciline (**110**)					[49]											
3,4-dihydro-3-hydroxygraciline (**111**)					[53]											
**Montanine type**																
pancracine (2-demethoxymontanine) (**112**)						[44,45]										
**Ismine type**																
ismine (**113**)		[32]	[17,18]	[29]	[24,35]			[17,19,21,22,34,42]	[24]				[30]			[23]
*N*-formylismine (**114**)											[49]					
**Miscellaneous types**																
apogalanthamine (**115**)			[19]													
gracilamine (**116**)					[54]											
tyramine (**117**)			[17]					[17]						[27]		
methyltyramine (**118**)			[17,19]					[17]								
*N*-feruloyltyramine (**119**)	[25]		[19,39]										[26]			
hordenine (**120**)	[25]		[17,18,19,22,39,41,42]	[28,29]			[24]	[17,22]			[14]		[30]			
stylopine (**121**)														[27]		
protopine (**122**)														[27]		
1-acetyl-β-carboline (**123**)			[18]				[24]						[26,30]			
salsoline (**124)**															[33]	
digracine (**125**)					[32]											
capnoidine (**126**)		[38]														
bulbocapnine (**127**)		[38]														

### What Is New?

The latest major review on alkaloid patterns in *Galanthus* spp. was published in early 2012, which poses the need for an update [20]. Since then, various new data have been collected on this topic. Four new species have been accepted: *G. bursanus*, *G. lebedevae*, *G. panjutinii* and *G. samothracicus*. Four species have been added to the list of phytochemically analyzed snowdrops: *G. fosteri*, *G. peshmenii*, *G. platyphyllus* and *G.* × *valentinei*. The classification of Amaryllidaceae alkaloids has undergone improvements, leading to the differentiation of 18 structural types compared to 10 in 2012. Since 2012, several new alkaloids were found in *Galanthus* and were characterized by NMR: 1-*O*-acetyl-9-*O*-methylpseudolycorine (**30**) from *G. woronowii* [33]; lycorine *N*-oxide (**32**) and incartine *N*-oxide (**36**) from *G. rizehensis* [24]; and oxoincartine (**35**), 9-*O*-demethyl-11-acetoxymaritidine (**57**) and 3-*O*-acetyl-9-*O*-demethyl-11-acetoxymaritidine (**60**) from *G. fosteri* [29]. In the past decade, an additional forty-three known alkaloids have been reported for the genus *Galanthus*:

- Norbelladine-type–*O*,*N*-dimethylnorbelladine (**2**);
- Lycorine-type–11,12-dehydrolycorene (**3**), assoanine (**5**), oxoassoanine (**6**), 2,11-didehydro-2-dehydroxylycorine (**7**), kirkine (fortucine) (**8**), hippadine (pratorine) (**13**), didehydroassoanine (**14**), 2-methoxypratosine (**15**), hippamine (**18**), 2-*O*-acetyllycorine (aulamine) (**19**), acetylcaranine (**22**), pluviine (**23**), pseudolycorine (**24**), 9-*O*-methylpseudolycorine (**25**), 1-methylplyviine (**27**), 1-acetylpluviine (**28**), 1-acetylnorpluviine (**29**), 1-*O*-acetyl-9-*O*-methylpseudolycorine (**30**), sternbergine (**31**), lycorine *N*-oxide (**32**), oxoincartine (**35**) and incartine *N*-oxide (**36**);
- Homolycorine-type–2α-methoxyhomolycorine (**43**);
- Galanthindole-type–9-*O*-demethyllycosinine B (**52**);
- Haemanthamine-type–9-*O*-demethylmaritidine (**56**), 9-*O*-demethyl-11-acetoxymaritidine (**57**), 3-*O*-acetyl-9-*O*-demethyl-11-acetoxymaritidine (**60**), haemanthidine (**64**) and 11,3′-*O*-(dihydrobutyryl)hamayne (**72**);
- Narciclasine-type–bicolorine (**75**);
- Galanthamine-type–*O*-acetylgalanthamine (**85**), 3-*O*-(3′-acetoxybutanoyl)galanthamine (**88**), lycoraminone (**89**), *N*-norlycoramine (*N*-demethyllycoramine) (**90**) and 3,4-anhydrogalanthamine (**92**);
- Pretazettine-type–tazettinol (**94**), deoxypretazettine (**98**) and pretazettine (**99**);
- Montanine-type–pancracine (2-demethoxymontanine) (**112**);
- Miscellaneous-type–apogalanthamine (**115**), 1-acetyl-β-carboline (**123**) and salsoline (**124**).

## 4. A Brief Overview of the Main Biological Activities of Amaryllidaceae Alkaloids Found in *Galanthus* spp.

### 4.1. Acetylcholinesterase Inhibitory Activity

Galanthamine (**81**) is an acetylcholinesterase (AChE) inhibitor used to alleviate the symptoms of Alzheimer’s disease (the disease itself is incurable) and other neurodegenerative diseases. It is implemented in the drugs Razadyne™, Reminyl™ and Nivalin^®^, and its usage was approved by the USA’s Food and Drug Administration (FDA) in 2001 [55]. Galanthamine’s potent inhibition on AChE, its natural origin, its ability to cross the blood–brain barrier and its fewer side effects make it preferable to other medications [56]. In Bulgaria, *G. nivalis* was originally used for the production of galanthamine in the early 1960s but was later replaced by *Leucojum aestivum* L., which was found to yield larger quantities suitable for the needs of the pharmaceutical industry [57]. Galanthamine is also the base of synthetic and hybrid molecules used in the search of novel AChE inhibitors [58,59].

Phytochemically analyzed *Galanthus* spp. have been thoroughly examined for their AChE inhibitory activity. Several articles have previously reported potent AChE inhibition for alkaloid extracts from *G. cilicicus*, *G. elwesii*, *G. fosteri*, *G. gracilis*, *G. rizehensis*, *G. krasnovii* and *G. woronowii* (IC_50_ values around and lower than 10 µg/mL) [19,24,29,30,31,32,35]. The alkaloid patterns of these extracts reveal that not only galanthamine, but also some haemanthamine-, lycorine- and homolycorine-type alkaloids may contribute to AChE inhibition.

### 4.2. Antimicrobial Activity

*Galanthus* extracts were tested against microorganisms and viruses on several occasions. For example, the alkaloid extract from *G. elwesii* showed moderate to no inhibition, with an MIC > 512 µg/mL for *Escherichia coli* and *Staphylococcus aureus*, 256 µg/mL for *Lodderomyces elongisporus*, 512 µg/mL for *Candida glabrata*, 1024 µg/mL for *C. albicans* (type strain) and *C. dubliniensis* and an MIC > 1024 µg/mL for *Candida albicans* (clinical isolates) [48]. In other instances, however, the methanolic or ethanolic plant extracts were assessed, but these are not mentioned here because no fractionation or isolation of alkaloids was performed. Overall, the information available on the antimicrobial properties of *Galanthus* spp. is scarce.

#### 4.2.1. Antibacterial Activity

The lycorine type is the most common in the assessment of antibacterial activity. The results for lycorine (**17**), its main representative, appear to be mixed, but it was mostly reported as weak or inactive against tested bacteria (*Agrobacterium tumefaciens*, *E. coli*, *Pseudomonas aeruginosa* and *S. aureus*). Weak to no inhibition was measured for 2-dehydroxy (caranine and acetylcaranine) and A-ring-modified (galanthine and 2-*O*-methylpseudolycorine) lycorine derivatives [60].

Naturally occurring acetyl and synthetic benzoyl lycorine derivatives were particularly active against *Flavobacterium columnare*, a well-known fish pathogen (MIC between 3 and 56 µg/mL).

C-ring unsaturated lycorine derivatives such as ungeremine (**9**) stand out as main antibacterial alkaloids. They have demonstrated strong inhibition of *E. coli*, *Edwardsiella ictaluri*, *F. columnare*, *Proteus mirabilis*, *P. aeruginosa*, *Enterococcus faecalis*, *Rhodococcus fascians* and *S. aureus* [60,61].

Gram-negative bacteria such as *E. coli*, *Neisseria gonorrhoeae*, *P. aeruginosa* and *Shigella flexneri* were found to be strongly inhibited by naturally occurring and synthetic narciclasine-type alkaloids (MIC values between 4 and 64 µg/mL), whereas moderate to weak inhibition was observed for *A. tumefaciens* and *S. aureus* [60].

Most of the tested homolycorine, crinine, haemanthamine-, pretazettine- and galanthamine-type alkaloids exhibited weak or no antibacterial properties against *E. coli*, *Helicobacter pylori*, *Klebsiella pneumoniae*, *P. aeruginosa*, *Bacillus subtilis* and *S. aureus*. Buphanidrine and distichamine are the two exceptions here, with an MIC of 63 µg/mL for *E. coli*, *K. pneumoniae* and *S. aureus* and an MIC of 130 µg/mL for *B. subtilis* [60].

#### 4.2.2. Antifungal Activity

From what is known in data in the literature, it can be surmised that Amaryllidaceae alkaloids are mostly inactive against yeasts and fungi (with minimum inhibitory concentrations (MICs) often > 200 µM) [62]. However, there are a few exceptions.

Yeasts from the genus *Candida* (primarily *C. albicans*) were found to be inhibited by lycorine (**17**), hippeastrine (**38**), vittatine (**61**), 11-hydroxyvittatine (**62**) and pancracine (**112**). Narciclasine- and lycorine-type alkaloids stand out as more effective against a broader spectrum of yeasts, including *Cryptococcus neoformans*, *Saccharomyces cerevisiae* and *L. elongisporus* [62].

Shen et al., 2014, previously reported the antifungal potential of lycorine (**17**). The alkaloid inhibited the growth of crop pathogenic fungi (including but not limited to the genera *Alternaria*, *Colletotrichum* and *Fusarium*) by 3.6–78.1%. *Fusarium graminearum*, which causes major losses to wheat and barley crops globally, was substantially inhibited by 74.7% [63].

When comparing lycorine to other inactive lycorine-type alkaloids, the C3-C4 double bond, two hydroxyl groups and one methylenedioxy group appear to be crucial for its antifungal activity. Lycorine blocks protein biosynthesis during cell division, and furthermore, at higher concentrations, it causes significant cell damage and the development of necrosis. These effects are also fundamental for its cytotoxic effects in cancer cells [62].

More recent works confirm these findings about the antifungal properties of Amaryllidaceae alkaloids against yeasts and phytopathogenic fungi [64,65].

#### 4.2.3. Antiviral Properties

Lycorine (**17**) is the best known Amaryllidaceae alkaloid to exhibit antiviral properties. It has been reported to affect coronaviruses (SARS-CoV, SARS-CoV-2 and MERS-CoV, with EC_50_ values between 0.01 and 2.12 µM), flaviviruses (Zika virus, Dengue virus and Duck Tembusu virus, with EC_50_ values between 0.22 and 2 µM), togaviruses (Chikungunya virus, Sindbi virus, Semliki forest virus and Venezuelan equine encephalitis virus, with EC_50_ values between 0.31 and 1.05 μM), picornaviruses (71–75% inhibition at 2.5 µg/mL), hepatitis C virus (EC_50_ = 0.32 µM) and bunyaviruses (Punta Toro, Rift Valley fever and Sandfly fever Sicilian). In most cases, a cytotoxic concentration of lycorine larger than 5 µM was measured [10,66,67,68,69]. Lycorine also possesses high antiretroviral (HIV-1) activity accompanied with low therapeutic indices. Its activity was found to be due to the inhibition of multiplication and not to the direct inactivation of extracellular viruses, and the mechanism of the antiviral effect was partially explained as the blocking of viral DNA polymerase [69].

Pretazettine (**99**) strongly inhibits the activity of reverse transcriptase from various oncogenic viruses by binding to the enzyme. This alkaloid has also been shown to be active against selected RNA-containing flaviviruses (Japanese encephalitis, yellow fever, Zika and Dengue) and bunyaviruses (Punta Toro and Rift Valley fever). It also possesses pronounced activity against Herpes simplex type 1 virus [67,69].

Flaviviruses (Dengue and Zika) were also observed to be inhibited by hippeastrine (**38**), haemanthamine (**63**), haemanthidine (**64**) and pancracine (**112**) (with EC_50_ values between 0.34 and 3.62 μM and CC_50_ values larger than 10 µM in most cases) [10].

Narciclasine-type alkaloids have been previously reported to inhibit flaviviruses (Japanese encephalitis, yellow fever and Dengue), with IC_50_ values between 0.03 and 2.8 μg/mL and CC_50_ values within 10-fold of the IC_50_ values. They also showed significant antiviral activity against bunyaviruses (Punta Toro and Rift Valley fever) [67].

## 5. Cytotoxicity and Anticancer Activity of Amaryllidaceae Alkaloids from *Galanthus* spp.

Cancer is one of the gravest threats to human health. In 2018, there were an estimated 18 million new cases of cancer and 10 million deaths from cancer worldwide. The predicted global burden will double to about 29–37 million new cancer cases by 2040. Of the 15 million deaths in individuals between the ages of 30 and 69 in 2018, 4.5 million were due to cancer [70]. The International Classification of Diseases (revision 11) lists more than 600 types of cancer, most of which require unique diagnostic and management approaches. The three most frequently occurring cancer types are breast, lung and prostate cancers. One-third to one-half of cancer cases can be prevented by reducing exposure to known risk factors. Most countries do not fully implement cancer prevention policies and programs, resulting in millions of avoidable cancer cases [70]. Thankfully, currently, cancer treatment offers options such as chemotherapy, radiotherapy, immunotherapy, targeted therapy, hormone therapy, stem cell transplant and surgery. Around 60% of successfully used cancer drugs available on the market are based on natural products, for example, taxol (from *Taxus brevifolia* Nutt.) and campthothecin (from *Camptotheca acuminata* Decne.) [71]. All of the above-mentioned statistics motivate the unstopping search for novel anticancer molecules among plants and other natural resources. Cytotoxicity is directly related to antitumor activity, and ideally, a tumor-suppressing drug would not affect normal eukaryotic cells in the organism.

Antitumor activity in Amaryllidaceae alkaloids was assessed as early as in the 1950s, when lycorine stood out in a research study on mice with sarcoma [72]. In the 1980s, certain alkaloids, for example, pancratistatin and narciclasine, gained attention due to their remarkable cytotoxic properties [73]. The work of Weniger et al. in 1995 marked significant progress towards the evaluation of the cytotoxicity of multiple Amaryllidaceae alkaloids of different structures on human and murine cell lines [74]. Since then, the main principles in the cytotoxicity of Amaryllidaceae have been established, including the mechanisms of action and structure–activity relationships. The lycorine and narciclasine types are the most studied and remain principal anticancer molecules.

During in vitro assays, the cytotoxic activity of samples is defined with the half-maximum inhibitory concentration (IC_50_). Based on the IC_50_ values, a suggested approximate scale for degrees of cytotoxicity for compounds would look like the following: 0 < IC_50_ < 10 µM (potent/active), 11 < IC_50_ < 30 µM (mild/moderate), 31 < IC_50_ < 50 µM (weak) and IC_50_ > 50 µM (inactive). For extracts, the same scale could be used with µg/mL.

### 5.1. Antitumor Potential of Galanthus spp. Extracts

Information on the anticancer and cytotoxic properties of *Galanthus* extracts is scarce. Jokhadze et al., 2007 previously described the cytotoxicity of several methanolic extracts from snowdrops [75]. Their experiment involved three human tumor cell lines, namely HCT-116, HeLa and HL-60, and extracts from eight *Galanthus* species derived from either aerial parts or bulbs. The results are heterogenous, but some main conclusions were made. Extracts from bulbs were more cytotoxic than those from aerial parts, and HCT-116 cells were the most sensitive of the three. The bulb extracts from four species showed remarkable activity towards the following cancer cells: *G. woronowii*, *G. krasnovii*, *G. alpinus* and *G. shaoricus* (=*G. alpinus* var. *alpinus*). They achieved IC_50_ values below 25 µg/mL and as low as 5.8–8.9 µg/mL for HCT-116 cells. Galanthamine, tazettine and lycorine were also tested against the three cell lines; the first two were deemed inactive with IC_50_ values > 100 µM, and lycorine was expectedly highly cytotoxic with IC_50_ values between 3.1 and 9.3 µM. The authors noted that lycorine was present in the tested *Galanthus* extracts but in concentrations in the range of 0.01–0.1%, so their cytotoxicity could not be contributed fully to this alkaloid [75].

### 5.2. Cytotoxicity and Anticancer Properties of Amaryllidaceae Alkaloids by Structural Type

No information was found about the cytotoxic or antitumor activity of galasine-, galanthindole-, plicamine-, secoplicamine- and graciline-type alkaloids previously reported in *Galanthus* spp.

Members of the norbelladine type have been previously examined for their cytotoxicity. The results for 4-*O*-methylnorbelladine (**1**) and 4-*O*,*N*-dimethylnorbelladine (**2**) revealed poor cytotoxic activity against both tumor and non-tumor cells [76].

Ismine (**113**) has been previously examined for its cytotoxicity, demonstrating poor cytotoxic activity against several tumor cell lines (IC_50_ > 30 µM) [76].

From what is known in the data in the literature, pretazettine-type alkaloids demonstrate diverse levels of cytotoxicity towards cancer and normal cells. Tazettine (**96**) and 3-*epi*-macronine (**104**) are primarily inactive, with IC_50_ values often above 25 μM [74,75,77,78,79,80]. The results for pretazettine (**99**) show good to moderate activity towards several cell lines, with IC_50_ values between 0.9 and 8.85 μM, with one exception (IC_50_ > 50 μM for Hep G2 cells) [74,81].

Pretazettine may exhibit cytotoxicity through inhibition on protein biosynthesis, by inducing DNA damage and by affecting the p-glycoprotein ABCB1 (adenosine triphosphate (ATP)-binding cassette transporter) [82]. Pretazettine’s antiproliferative effects have been previously shown in both human and murine cell lines [81], and while the exact molecular mechanisms underlying its cytotoxicity are still being investigated, its ability to target multiple cellular processes makes it a promising candidate for cancer therapy.

Various montanine-type alkaloids have been previously studied for their antitumor and cytotoxic potential. In 2002, Labraña et al. reported that pancracine (**112**) was inactive against normal murine L6 myoblasts (IC_50_ > 90 µg/mL) [83]. Later, Breiterová et al., 2020, determined that this alkaloid was strongly cytotoxic to eight tumor cell lines and one normal cell line, with IC_50_ values between 2.20 and 5.15 µM. The largest of these values (5.15 µM) was observed for the normal cell line and was far less toxic than the positive control doxorubicin [84].

Hordenine (**120**) is a main tyramine-type alkaloid found in several *Galanthus* species. Zhang et al., 1980, conducted research involving mice transplanted with S_180_ sarcoma and found that a 10-day treatment with hordenine at a dose of 50 mg/kg per day was able to reduce tumor weight by 50% [85].

Akhil et al., 2020, analyzed the effects of a methanolic extract of germinated *Hordeum vulgare* seeds on triple-negative breast cancer cells. Their findings demonstrate significant cytotoxicity in said extract. Hordenine was found as a main constituent of the extract and was determined as the most probable antitumor compound [86].

Hordenine was the subject of an anticancer assay where two human lung cancer cell lines and one human normal kidney cell line were used. It exhibited good cytotoxicity against the cancer cell lines (IC_50_ values of 14.95 and 21.30 µM) but did not affect normal cells (IC_50_ > 200 µM) [87].

Hordenine was found to exhibit cytotoxicity through apoptosis induction and pyruvate dehydrogenase kinase 3 (PDK3) inhibition (disruption of glucose metabolism regulation) [86,87].

Narciclasine-type alkaloids are not common for the genus *Galanthus*. Trisphaeridine is the most often found alkaloid of this structural type, followed by 5,6-dihydrobicolorine and arolycoricidine. Some of the most potently cytotoxic alkaloids, such as narciclasine and pancratistatin, have not yet been confirmed in *Galanthus* spp.

Trisphaeridine (**74**) and 5,6-dihydrobicolorine (**76**) and their derivatives were found to be inactive in cytotoxicity assays (IC_50_ > 20 µM) [78,81,88]. There is one exception available: 5,6-dihydrobicolorine was active against human T-lymphoma Molt4 cells with an IC_50_ value of 0.8 µg/mL, although in the same research, it was cytotoxic to normal murine alveolar fibroblasts with an IC_50_ value of 0.7 µg/mL [74]. Bicolorine (**75**) exhibited cytotoxic activity against human prostate cancer PC-3 cells with an IC_50_ value of around 15 µM [76].

In narciclasine-type alkaloids, a combination of structural functionalities is crucial for cytotoxicity: A-ring methylenedioxyphenyl; B-ring lactam; partial or complete saturation of the C-ring; and the presence of C-ring hydroxyl groups. When comparing trisphaeridine to bicolorine, the added *N*-methyl group in the latter enhances cytotoxicity. Saturation of the B-ring in bicolorine yielding 5,6-dihydrobicolorine has the same effect [76].

Narciclasine inhibits the 60S subunit of ribosomes and thus protein biosynthesis, targets translation elongation factor eEF1A, triggers actin stress fiber formation via the activation of the small GTPase RhoA (causing cytoskeleton changes diminishing mitotic rates) and induces selective apoptosis in tumor cells [89].

Lycorine-type alkaloids are well studied when it comes to cytotoxicity and anticancer research (Table S1). Lycorine’s (**7**) antitumor properties were first discovered in 1976 by the Spanish scientist A. Jimenez when he found that lycorine could inhibit translation (protein biosynthesis) in eukaryotic cells. Lycorine’s partially saturated C-ring is crucial for its antitumor activity together with the hydroxyl groups at C1 and C2 because of their role in the formation of hydrogen bonds. This was deduced after a dehydroxy lycorine analog was tested against several cell lines and showed very weak activity compared to lycorine itself [90].

Lycorine has been proven to be selective towards malignant cells compared to normal eukaryotic cells. At concentrations critical for tumors, it can only weakly and reversibly affect normal cells. The alkaloid is highly cytotoxic towards tumor cells in low concentrations. The mechanisms for its antitumor activity include induced apoptosis, cell cycle arrest, induced necrosis and the inhibition of autophagy [91]. It can also be cytotoxic to apoptosis-resistant tumor cells [92].

Pseudolycorine (**24**), lycorine’s methylenedioxy analog, is another highly cytotoxic alkaloid with an IC_50_ value often below 10 µM, but it is less active than lycorine itself. Other modifications in the A- and C-rings of lycorine or pseudolycorine (additions of acetoxy, methoxy groups and dehydroxylation) appear to worsen the cytotoxic effects of these molecules, as seen in caranine, acetylcaranine, 2-*O*-acetyllycorine, galanthine and sternbergine [90].

Ungeremine (**9**) and hippadine (**13**) are lycorine derivatives with a partially unsaturated C-ring. Ungeremine was proven to be highly cytotoxic in several cases and is a molecule of increased interest. Its activity might be enhanced by the additional B-ring aromatic moiety. Contrastingly, hippadine with its 6-oxo additional substitution and D-ring unsaturation was inactive in anticancer assays. Incartine (**34**), a methoxylated, C-ring-saturated pseudolycorine derivative, was also determined to be a poor cytotoxic agent [90].

Several homolycorine-type alkaloids have been proven to possess good to moderate cytotoxic properties (Table S2) [93]. When comparing the results for homolycorine (**40**) and masonine (**37**), it seems that the change in the A-ring from two methoxy groups to a methylenedioxy one does not improve cytotoxicity. Demethylation at C8 of the A-ring yields 8-*O*-demethylhomolycorine (**41**), an alkaloid which was shown to be far more effective than homolycorine at inhibiting the growth of Molt4 cells (IC_50_ = 18.5 μg/mL versus > 50 μg/mL, respectively). However, both compounds had a poor influence on HepG2 cells (IC_50_ > 50 μg/mL) [74]. More information is needed on the cytotoxicity of 8-*O*-demethylhomolycorine, as well as 9-*O*-demethylhomolycorine, to determine whether activity could be influenced by the elimination of either methoxy group. In hippeastrine (**38**), the hydroxyl group present at C2 of the C-ring is an important structural feature, since its dehydroxy counterpart, masonine, was proven to be a poor cytotoxic agent [93].

Several articles suggest that homolycorine-type alkaloids may exhibit cytotoxicity through interactions with the cytoskeleton of tumor cells or the inhibition of topoisomerase I, whose activity is increased in rapidly proliferating cells [93].

Crinine- and haemanthamine-type alkaloids are phenanthridine molecules with a 5,10b-ethano bridge, which is β-oriented in the former and α-oriented in the latter. Available information on the cytotoxicity of these structures suggests that the orientation of the bridge is a key component of activity, since haemanthamine-type alkaloids are more cytotoxic than crinine [94].

Crinine (**53**) and buphanisine (**54**) have been previously tested against a total of 17 tumor and normal cell lines but showed poor cytotoxic ability with IC_50_ values of over 50 μM in most cases (Table S3).

Alkaloids with an α-ethano bridge included in articles researching cytotoxicity include haemanthamine (**63**), haemanthidine (**64**), hamayne (**65**), vittatine (**61**), 11-hydroxyvittatine (**62**) and 8-*O*-demethylmaritidine (**55**). Haemanthamine and haemanthidine show the best results in this group, with IC_50_ values below 5 μM in many cases (Table S4). Some articles report selectivity towards cancer cells compared to normal ones. The additional hydroxyl group in haemanthidine might be the reason for the slightly higher IC_50_ values of this compound. It is worth noting that alkaloids with the C3-methoxy group (haemanthamine, haemanthidine and crinamine) achieve better results in cytotoxicity assays than those with the C3-hydroxyl group (8-*O*-demethylmaritidine, hamayne and vittatine) [94].

Haemanthamine’s cytotoxicity is mainly due to the induction of apoptosis (cell cycle arrest, the attenuation of mitochondrial membrane potential and nucleolar stress and the inhibition of ribosome biogenesis), the inhibition of protein synthesis by interaction with the 60S subunit of ribosomes or through direct interaction with DNA [94,95].

Cripowellin-type alkaloids have not been reported in *Galanthus*; however, it is important to mention them here because of the remarkable cytotoxicity they demonstrate (IC_50_ values in the nM scale) [96]. Their structural similarity to haemanthamine further solidifies the idea that the α-ethano bridge is crucial, but glycosylation at C3 might also help enhance cytotoxicity since such modification is not observed in haemanthamine-type alkaloids.

Galanthamine-type alkaloids, despite being renowned mostly for their anticholinesterase properties, have been fairly well incorporated in anticancer research. The results show that they are mostly inactive or poorly cytotoxic, with minor exceptions (Table S5). The available results suggest that the *N*-demethylation of galanthamine (**81**) yielding *N*-norgalanthamine (**82**) could be beneficial towards cytotoxicity by increasing the charge around the nitrogen atom. The oxidation of the *N*-methyl group also enhances cytotoxicity but to a lesser extent, since *N*-formylnorgalanthamine (**83**) showed poorer activity than *N*-norgalanthamine. In alkaloids such as galanthamine *N*-oxide and *N*-chloromethylgalanthamine, charge is being pulled away from the nitrogen atom, leading to inactivity in cytotoxicity assays. Several modifications in the C-ring of galanthamine did not improve cytotoxic properties: the substitution of the hydroxyl group (in chlidanthine) and the reduction in the double bond (in lycoramine (**91**)). The oxidation of the hydroxyl group (in narwedine (**79**)) could benefit cytotoxicity [76].

## 6. Conclusions and Future Possibilities

In the past decade, great advancements have been made towards the taxonomical and phytochemical characterization of *Galanthus* spp. (snowdrops). These plants are a source of immense variability of Amaryllidaceae alkaloids, some of which possess unique structural features. Still, not every *Galanthus* sp. has been studied for its alkaloid patterns. No information is available on nine out of twenty-five known species. Several research articles include compounds that have been detected but left unidentified. The structural diversity within Amaryllidaceae alkaloids translates to a wide spectrum of biological and pharmacological activities. In this regard, compounds found exclusively in *Galanthus* spp. are either understudied or not studied at all, especially when it comes to cytotoxicity. The influence of Amaryllidaceae alkaloids on cellular pathways such as PI3K/Akt/mTOR (cell growth and survival), RAS/RAF/MEK/ERK (cell division and differentiation), p53 (apoptosis) and WNT/β-catenin (cell-to-cell communication and the regulation of gene expression) could be assessed to gain more insights into the mechanisms of action. These gaps in the knowledge about snowdrops, their alkaloid patterns and possible applications enable many exciting opportunities for future investigations.

## Figures and Tables

**Table 1:** Geographical distributions of *Galanthus* spp. according to Plants of the World Online [1].

Species	Countries Where the Species Grows Naturally	Countries Where the Species Is Introduced
*G. alpinus* Sosn.	Armenia, Azerbaijan, Georgia, Russia (North Caucasus), Turkey (Asian)	Germany
*G. angustifolius* Koss	Russia (North Caucasus)	
*G. bursanus* Zubov, Konca & A.P.Davis	Turkey (Asian)	
*G. cilicicus* Baker	Turkey (Asian)	
*G. elwesii* Hook.f.	Albania, Bosnia and Herzegovina, Bulgaria, Croatia, Greece (incl. East Aegean Islands), Kosovo, Montenegro, North Macedonia, Serbia, Slovenia, Turkey (Asian), Ukraine	Austria, Germany, Great Britain, Ireland, USA (New York, Ohio, Pennsylvania)
*G. fosteri* Baker	Israel, Jordan, Lebanon, Palestine, Syria, Turkey (Asian)	Germany
*G. gracilis* Čelak.	Bulgaria, Romania, Turkey (Asian), Ukraine, Greece (doubtful)	Germany
*G. ikariae* Baker	Greece (incl. East Aegean Islands)	Germany
*G. koenenianus* Lobin, C.D.Brickell & A.P.Davis	Turkey (Asian)	
*G. krasnovii* Khokhr.	Armenia, Azerbaijan, Georgia, Turkey (Asian)	
*G. lagodechianus* (*kemulariae*) Kem.-Nath.	Armenia, Azerbaijan, Georgia, Russia (North Caucasus)	
*G. lebedevae* Timukhin & Tuniyev	Russia (North Caucasus)	
*G. nivalis* L.	Albania, Austria, Belarus, Bosnia and Herzegovina, Bulgaria, Croatia, Czech Republic, France, Germany, Greece, Hungary, Italy, Kosovo, Montenegro, North Macedonia, Poland, Romania, Serbia, Sicilia, Slovakia, Slovenia, Spain, Switzerland, Turkey (European), Ukraine, Yugoslavia	Belgium, Canada (New Brunswick, Newfoundland, Ontario), Great Britain, Ireland, Netherlands, Norway, Sweden, USA (Maryland, Massachusetts, Michigan, New Jersey, New York, North Carolina, Ohio, Pennsylvania, Utah, Virginia, Washington)
*G. panjutinii* Zubov & A.P.Davis	Armenia, Azerbaijan, Georgia, Russia (North Caucasus)	
*G. peshmenii* A.P.Davis & C.D.Brickell	East Aegean Islands, Turkey (Asian)	
*G. platyphyllus* (*latifolius*) Traub & Moldenke	Armenia, Azerbaijan, Georgia, Russia (North Caucasus)	
*G. plicatus* M.Bieb.	Krym, Romania, Russia (North Caucasus), Turkey (Asian), Ukraine	Germany, Great Britain, Ireland, USA (Alabama)
*G. reginae-olgae* Orph.	Bosnia and Herzegovina, Croatia, Greece, Italy (Sicily), Kosovo, Montenegro, North Macedonia, Serbia, Slovenia	
*G. rizehensis* Stern	Armenia, Azerbaijan, Georgia, Russia (North Caucasus), Turkey (Asian)	Germany
*G. samothracicus* Kit Tan & Biel	Greece	
*G. transcaucasicus* Fomin.	Armenia, Azerbaijan, Georgia, Iran	
*G. trojanus* A.P.Davis & Özhatay	Turkey (Asian)	
*G. woronowii* Losinsk.	Armenia, Azerbaijan, Georgia, Russia (North Caucasus), Turkey (Asian)	Austria, Germany, Great Britain
*G.* × *allenii* Baker	Armenia, Azerbaijan, Georgia	
*G.* × *valentinei* Beck	Turkey (Asian, European)	Great Britain, Ireland

G. × *allenii* and G. × *valentinei* are naturally occurring hybrids (× *allenii* = *alpinus* × *woronowii*, × *valentinei* = *plicatus* × *nivalis*).

## Data Availability

No new data were created or analyzed in this study.

## Supplementary Materials

The following supporting information can be downloaded at: https://www.mdpi.com/article/10.3390/plants13243577/s1. References [97,98,99,100,101,102,103,104,105,106,107,108,109,110,111,112] are cited in the supplementary materials.
