# Supplementary material for: Phytochemical and Cytotoxic Aspects of Amaryllidaceae Alkaloids in Galanthus Species: A Review
Source: Plants (Basel). 2024 Dec 21;13(24):3577. doi: 10.3390/plants13243577 (PMC11678157; doi:10.3390/plants13243577)
Supplement: Supplementary file 1 [file plants-13-03577-s001.zip › plants-3367649-supplementary.pdf]

	A-431	A549	A2780	AGS	BCA-1	BT-549	Caco-2	CEM	COL-1	COLO-201	G-361	H1299	HCT 15	HCT 116	HeLa	Hep G2	HL-60	Hs578T	HSC-2	HT-29	HT-1080	Jurkat	K562	KB	KB-V1	LNCaP	LoVo	Reference
ungeremine (**9**)		0.43*															0.63*										13.1*	[1]
dihydrolycorine (**11**)		> 20																									> 20	[2]
hippadine (**13**)		> 100															> 100										> 100	[1]
caranine (**16**)							64.4													46.6								[3]
lycorine (**17**)	1.3*	1.0; 1.9; 9.55; 0.65*	1.2	0.8; 4.17*	1.6*	1.1	0.99	1.6	0.8*	0.8	5.0	0.9		3.1; > 50	0.9; 9.3; 10.6	3.7; 13*	1.1; 1.3; 1.6; 8.2; 0.17*		5.1	0.8; 1.2; 3.2	0.5*	1.3	2.3; 3.6	0.3*	0.4*	0.5*	0.59; 17.36	[1,2, 3–14]
2-*O*-acetyllycorine (**19**)	14.05														10.31													[15]
acetylcaranine (**22**)							29.5													19.2								[3]
pseudolycorine (**24**)		3.97												0.85; 4.6	5.0	10*	1.10										8.98	[1,5,12, 16,17]
galanthine (**26**)		> 20					> 10								> 25	> 50				> 10							> 20	[2,5, 10, 16]
sternbergine (**31**)																> 50												[5]
incartine (**34**)		> 50											> 50					> 50										[18]

Table S1. Cytotoxicity of lycorine type alkaloids found in *Galanthus* spp. towards cancer and normal cell lines. All values in µM, except when noted with * which means µg/ml. A-431 = human skin epidermoid carcinoma, A549 = human lung carcinoma, A2780 = human ovarian carcinoma, AGS = human gastric adenocarcinoma, BCA-1 = human breast adenocarcinoma, BT-549 = human breast ductal carcinoma, Caco-2 = human colorectal adenocarcinoma, CEM = human acute T-cell leukemia, COL-1 = human colon carcinoma, COLO-201 = human colorectal adenocarcinoma, G-361 = human malignant melanoma, H1299 = human non-small cell lung carcinoma, HCT 15 = human colon adenocarcinoma, HCT 116 = human colorectal carcinoma, HeLa = human cervix adenocarcinoma, Hep G2 = human hepatoma, HL-60 = human acute promyelocytic leukemia, Hs578T = human breast carcinoma, HSC-2 = human oral squamous cell carcinoma, HT-29 = human colorectal adenocarcinoma, HT-1080 = human fibrosarcoma, Jurkat = human acute T cell leukemia, K-562 = human myeloid leukemia, KB = human oral epidermoid carcinoma, KB-V1 = vinblastine resistant KB, LNCaP = human hormone-dependent prostatic carcinoma, LoVo = human colorectal adenocarcinoma

	LUC-1	MCF7	MDA-MB-231	MDA-MB-468	MEL-2	MOLT-4	OVCAR8	PANC-1	SAOS-2	SF295	SK-MEL-28	SW-480	U-373	ZR-75-1	6T-CEM	L5178 MDR	L5178 PAR	P-388	Beas-2B	BJ	FHs 74 Int	HFF-1	MCF12A	NHDF	RPE	LMTK	Vero (E6)	Reference
ungeremine (**9**)															3.89*												> 100	[1,19]
dihydrolycorine (**11**)																												[2]
hippadine (**13**)															> 100													[1]
caranine (**16**)																					> 100							[3]
lycorine (**17**)	0.9*	0.7; 0.8; 13; > 50	0.9; 1.4	1.4	1.6*	1.0; 0.4*		0.7	1.2			0.9	0.3*	0.9*	1.42*			0.9*	4.9	1.9	22.7	1.3	3.0	0.8	> 50	0.3*	1.2	[1,2, 4–13,19]
2-*O*-acetyllycorine (**19**)		19.83														13.02	11.56											[15]
acetylcaranine (**22**)																					66.1							[3]
pseudolycorine (**24**)		10.6				0.5*	1.59			1.81															42.6	0.4*	3.41	[2,5,12, 17,20]
galanthine (**26**)						3.9*															> 10					48*		[2,5,10]
sternbergine (**31**)						13.0*																				7.0*		[5]
incartine (**34**)		> 50	> 50								> 50																	[18]

Table S1 (continued). Cytotoxicity of lycorine type alkaloids found in *Galanthus* spp. towards cancer and normal cell lines. All values in µM, except when noted with * which means µg/ml. LUC-1 = human lung carcinoma, MCF7 = human breast adenocarcinoma, MDA-MB-231 = human triple-negative breast adenocarcinoma, MDA-MB-468 = human triple-negative breast adenocarcinoma, MEL-2 = human melanoma, MOLT-4 = human acute lymphoblastic leukemia, OVCAR8 = human ovarian adenocarcinoma, PANC-1 = human pancreas epithelioid carcinoma, Saos-2 = human osteosarcoma, SF295 = human glioblastoma, SK-MEL-28 = human malignant melanoma, SW-480 = human colorectal adenocarcinoma, U-373 = human glioblastoma, ZR-75-1 = human hormone-dependent breast adenocarcinoma, 6T-CEM = human acute T-cell leukemia, L5178 MDR = multidrug resistant mouse lymphoma, L5178 PAR = native mouse lymphoma, P-388 = murine lymphoma, Beas-2B = human non-tumor bronchial epithelial cells, BJ = human non-tumor fibroblasts, FHs 74 Int = human non-tumor small intestine epithelial cells, HFF-1 = human non-tumor fibroblasts, MCF12A = human non-tumor breast cells, NHDF = human non-tumor dermal fibroblasts, RPE = human non-tumor retinal pigment epithelial cells, LMTK = murine non-tumor alveolar fibroblasts, Vero (E6) = monkey non-tumor kidney epithelial cells

	A549	A2780	CEM	DU145	G-361	HCT 116	HeLa	Hep G2	HL-60	HT-29	Huh7	Jurkat	K-562	LoVo	MCF7	MDA-MB-231	MOLT-4	PANC-1	Saos-2	L5178 MDR	L5178 PAR	BJ	MRC-5	LMTK	Vero (E6)	Reference
hippeastrine (**38**)	> 10	10		101.39		73.76	> 10; > 25	40*	13.2	> 10	87.05	< 10			> 10	24.7	10; 16.5*	> 10	> 10				> 10	0.4*	25	[5,7,13, 16,21,22]
homolycorine (**40**)	> 20		15		32.9		>30; > 50	> 50*	> 50				19.4	> 20	> 50		> 50*			69.13	28.51	20.8		0.5*		[2,5,7,9,15]
8-*O*-demethylhomo-lycorine (**41**)								> 50*								> 40	18.5*							0.8*		[5,7,13]

Table S2. Cytotoxicity of homolycorine type alkaloids found in *Galanthus* spp. towards cancer and normal cell lines. All values in μM, except when noted with * which means μg/ml. A549 = human lung carcinoma, A2780 = human ovarian carcinoma, CEM = human acute T-cell leukemia, DU145 = human prostatic carcinoma, G-361 = human malignant melanoma, HCT 116 = human colorectal carcinoma, HeLa = human cervix adenocarcinoma, Hep G2 = human hepatoma, HL-60 = human acute promyelocytic leukemia, HT-29 = human colorectal adenocarcinoma, Huh7 = human hepatocellular carcinoma, Jurkat = human acute T cell leukemia, K-562 = human myeloid leukemia, LoVo = human colorectal adenocarcinoma, MCF7 = human breast adenocarcinoma, MDA-MB-231 = human triple-negative breast adenocarcinoma; MOLT-4 = human acute lymphoblastic leukemia, PANC-1 = human pancreas epithelioid carcinoma, Saos-2 = human osteosarcoma, L5178 MDR = multidrug resistant mouse lymphoma, L5178 PAR = native mouse lymphoma, BJ = human non-tumor fibroblasts, MRC-5 = human non-tumor lung fibroblasts, LMTK = murine non-tumor alveolar fibroblasts, Vero (E6) = monkey non-tumor kidney epithelial cells

	A-431	BCA-1	Caco-2	COL-1	HeLa	HT-29	HT-1080	KB	KB-V1	LNCaP	LUC-1	MEL-2	U-373	ZR-75-1	P-388	FHs 74 Int	Vero (E6)	Reference
crinine (**53**)			64.5		> 50	50.8										>100		[3,23]
buphanisine (**54**)	> 20*	> 20*	8.6	> 20*	> 25; > 50	5.3	> 20*	> 20*	19.8*	> 20*	> 20*	> 20*	> 20*	> 20*	> 5*	22.8	> 25	[3,4,16,23]

Table S3. Cytotoxicity of crinine type alkaloids found in *Galanthus* spp. towards cancer and normal cell lines. All values in μM, except when noted with * which means μg/ml. A-431 = human skin epidermoid carcinoma, BCA-1 = human breast adenocarcinoma, Caco-2 = human colorectal adenocarcinoma, COL-1 = human colon carcinoma, HeLa = human cervix adenocarcinoma, HT-29 = human colorectal adenocarcinoma, HT-1080 = human fibrosarcoma, KB = human oral epidermoid carcinoma, KB-V1 = vinblastine resistant KB, LNCaP = human hormone-dependent prostatic carcinoma, LUC-1 = human lung carcinoma, MEL-2 = human melanoma, U-373 = human glioblastoma, ZR-75-1 = human hormone-dependent breast adenocarcinoma, P-388 = murine lymphoma, FHs 74 Int = human non-tumor small intestine epithelial cells, Vero (E6) = monkey non-tumor kidney epithelial cells

	A549	A2780	AGS	BT-549	Caco-2	CEM	COLO-201	G-361	H1299	HCT 116	HeLa	Hep G2	HL-60	HSC-2	HT-29	Jurkat	K-562	LoVo	Reference
8-*O*-demethylmaritidine (**55**)											> 25								[16]
vittatine (**61**)															21.91*				[24]
11-hydroxyvittatine (**62**)										35.71	> 25		> 50						[16,17]
haemanthamine (**63**)	1.1; 2.5; 4.48	0.7	1.0	1.0	0.99	2.1	1.0	3.7	1.2		0.6; 7.0; 8.3	4.8; >50*	0.9; 1.4; 1.6	28.4	0.59; 0.3; 2.1	1.4	2.5; 3.4	9.02	[2,5,7–11,23]
haemanthidine (**64**)	9.6; 11.36	2.3	1.5	9.6	3.3		9.3		9.4		1.6; 13.9		1.6; 2.0	13.3	1.7; 1.3	9.3		17.37	[2,7,10,11,23]
haemanthidine chloride	1.1											1.5	0.91		1.2		1.0		[8]
hamayne (**65**)					> 10										> 10				[10]

	MCF7	MDA-MB-231	MDA-MB-468	MOLT-4	NCI-H460	OVCAR8	PANC-1	RXF393	Saos-2	SF295	SW-480	Beas-2B	BJ	FHs 74 Int	HFF-1	MCF12A	MRC-5	LMTK	Vero (E6)	Reference
vittatine (**61**)					15.88*			29.57*												[24]
11-hydroxyvittatine (**62**)						> 50				> 50									10.57	[17,20]
haemanthamine (**63**)	0.8; 1.6; 8.1	3.9; 9.5	3.8	1.2; 1.2*			9.8		1.2		0.7	5.0	2.7	19.5	2.7	6.5	0.5	0.5*	0.76	[5,8-11,13,20]
haemanthidine (**64**)	1.8; 2.7	4.9; 8.5	3.5	1.7			9.7		9.7		1.7			11.6	2.7	5.0	1.4		5.43	[10,11,13,20]
haemanthidine chloride												3.7								[8]
hamayne (**65**)														> 10						[10]

Table S4. Cytotoxicity of haemanthamine type alkaloids found in *Galanthus* spp. towards cancer and normal cell lines. All values in μM, except when noted with * which means μg/ml. A549 = human lung carcinoma, A2780 = human ovarian carcinoma, AGS = human gastric adenocarcinoma, BT-549 = human breast ductal carcinoma, Caco-2 = human colorectal adenocarcinoma, CEM = human acute T-cell leukemia, COLO-201 = human colorectal adenocarcinoma, G-361 = human malignant melanoma, H1299 = human non-small cell lung carcinoma, HCT 116 = human colorectal carcinoma, HeLa = human cervix adenocarcinoma, Hep G2 = human hepatoma, HL-60 = human acute promyelocytic leukemia, HSC-2 = human oral squamous cell carcinoma, HT-29 = human colorectal adenocarcinoma, Jurkat = human acute T cell leukemia, K-562 = human myeloid leukemia, LoVo = human colorectal adenocarcinoma, MCF7 = human breast adenocarcinoma, MDA-MB-231 = human triple-negative breast adenocarcinoma, MDA-MB-468 = human triple-negative breast adenocarcinoma, MOLT-4 = human acute lymphoblastic leukemia, NCI-H460 = non-small cell lung carcinoma, OVCAR8 = human ovarian adenocarcinoma, PANC-1 = human pancreas epithelioid carcinoma, RXF393 = human renal cell carcinoma, Saos-2 = human osteosarcoma, SF295 = human glioblastoma, SW-480 = human colorectal adenocarcinoma, Beas-2B = human non-tumor bronchial epithelial cells, BJ = human non-tumor fibroblasts, FHs 74 Int = human non-tumor small intestine epithelial cells, MCF12A = human non-tumor breast cells, MRC-5 = human non-tumor lung fibroblasts, LMTK = murine non-tumor alveolar fibroblasts, Vero (E6) = monkey non-tumor kidney epithelial cells

	A549	A2780	Caco-2	CEM	G-361	HCT 116	HeLa	Hep G2	HL-60	HT-29	Jurkat	K-562	MCF7	MDA-MB-231	MOLT-4	OVCAR8	Saos-2	SF295	BJ	FHs 74 Int	MRC-5	LMTK	Reference
narwedine (**79**)				31.6	> 50		> 50					> 50	> 50						> 50				[9]
3-*epi*-galanthamine (**80**)														> 40									[13]
galanthamine (**81**)			> 50	> 50	> 50	> 50,> 100	> 50, > 100	> 50*	> 50, > 100	49.27		> 50	> 50		> 50*	> 50		> 50	> 50	> 50		0.5*	[5–7,9,10,17]
N-norgalanthamine (**82**)								> 50*							0.6*							0.5*	[5]
N-formylnorgalanthamine (**83**)								9.0*							1.6*							0.6*	[5]
sanguinine (**84**)						29.00			32.08							> 50		> 50					[17]
lycoraminone (**89**)	> 10	> 10					> 10			> 10	> 10		> 10		> 10		> 10				> 10		[25]
N-norlycoramine (**90**)	> 10	> 10					> 10			> 10	> 10		> 10		> 10		> 10				> 10		[25]
lycoramine (**91**)				> 50	> 50		> 50		> 50			> 50	> 50						> 50				[7,9]

Table S5. Cytotoxicity of galanthamine type alkaloids found in *Galanthus* spp. towards cancer and normal cell lines. All values in μM, except when noted with * which means μg/ml. A549 = human lung carcinoma, A2780 = human ovarian carcinoma, Caco-2 = human colorectal adenocarcinoma, CEM = human acute T-cell leukemia, G-361 = human malignant melanoma, HCT 116 = human colorectal carcinoma, HeLa = human cervix adenocarcinoma, Hep G2 = human hepatoma, HL-60 = human acute promyelocytic leukemia, HT-29 = human colorectal adenocarcinoma. Jurkat = human acute T cell leukemia, K-562 = human myeloid leukemia, MCF7 = human breast adenocarcinoma, MDA-MB-231 = human triple-negative breast adenocarcinoma, MOLT-4 = human acute lymphoblastic leukemia, OVCAR8 = human ovarian adenocarcinoma, Saos-2 = human osteosarcoma, SF295 = human glioblastoma, BJ = human non-tumor fibroblasts, FHs 74 Int = human non-tumor small intestine epithelial cells, MRC-5 = human non-tumor lung fibroblasts, LMTK = murine non-tumor alveolar fibroblasts

References:

1. Sun, Q.; Shen, Y.-H.; Tian, J.-M.; Tang, J.; Su, J.; Liu, R.-H.; Li, H.-L.; Xu, X.-K.; Zhang, W.-D. Chemical Constituents of *Crinum asiaticum* L. var. *sinicum* Baker and Their Cytotoxic Activities. *Chem Biodivers* **2009**, *6*, 1751–1757. https://doi.org/10.1002/cbdv.200800273
2. Ang, S.; Liu, X.M.; Huang, X.J.; Zhang, D.M., Zhang, W.; Wang, L.; Ye, W.C. Four New Amaryllidaceae Alkaloids from *Lycoris radiata* and Their Cytotoxicity. *Planta Med* **2015**, *81*, 1712–1718. https://doi.org/10.1055/s-0035-1557743
3. Vaněčková, N.; Hošťálková, A.; Šafratová, M.; Kuneš, J.; Hulcová, D.; Hrabinová, M.; Doskočil, I.; Štěpánková, S.; Opletal, L.; Nováková, L.; Jun, D.; Chlebek, J.; Cahlíková, L. Isolation of Amaryllidaceae alkaloids from *Nerine bowdenii* W. Watson and their biological activities. *RSC Adv* **2016**, 6, 80114–80120. https://doi.org/10.1039/C6RA20205E
4. Likhitwitayawuid, K.; Angerhofer, C.K.; Chai, H.; Pezzuto, J.M.; Cordell, G.A. Cytotoxic and Antimalarial Alkaloids from the Bulbs of *Crinum amabile*. *J Nat Prod* **1993**, *56*, 1331–1338. https://doi.org/10.1021/np50098a017
5. Weniger, B.; Italiano, L.; Beck, J.-P.; Bastida, J.; Bergoñon, S.; Codina, C.; Lobstein, A.; Anton, R. Cytotoxic Activity of Amaryllidaceae Alkaloids. *Planta Med* **1995**, *61*, 77–79. https://doi.org/10.1055/s-2006-958007
6. Jokhadze, M.; Eristavi, L.; Kutchukhidze, J.; Chariot, A.; Angenot, L.; Tits, M.; Jansen, O.; Frédérich, M. *In Vitro* Cytotoxicity of some Medicinal Plants from Georgian Amaryllidaceae. *Phytother Res* **2007**, *21*, 622–624. https://doi.org/10.1002/ptr.2130
7. Jitsuno, M.; Yokosuka, A.; Hashimoto, K.; Amano, O.; Sakagami, H.; Mimaki, Y. Chemical Constituents of *Lycoris albiflora* and their Cytotoxic Activities. *Nat Prod Commun* **2011**, *6*, 187–192. https://doi.org/10.1177/1934578X1100600208
8. Luo, Z.; Wang, F.; Zhang, J.; Li, X.; Zhang, M.; Hao, X.; Xue, Y.; Li, Y.; Horgen, F.D.; Yao, G.; et al. Cytotoxic Alkaloids from the Whole Plants of *Zephyranthes candida*. *J Nat Prod* **2012**, *75*, 2113–2120. https://doi.org/10.1021/np3005425
9. Nair, J.J.; Rárová, L.; Strnad, M.; Bastida, J.; van Staden, J. Apoptosis-inducing effects of distichamine and narciprimine, rare alkaloids of the plant family Amaryllidaceae. *Bioorg Med Chem Lett* **2012**, *22*, 6195–6199. https://doi.org/10.1016/j.bmcl.2012.08.005
10. Doskočil, I.; Hošťálková, A.; Šafratová, M.; Benešová, N.; Havlík, J.; Havelek, R.; Kuneš, J.; Královec, K.; Chlebek, J.; Cahlíková, L. Cytotoxic activities of Amaryllidaceae alkaloids against gastrointestinal cancer cells. *Phytochem Lett* **2015**, *13*, 394–398. https://doi.org/10.1016/j.phytol.2015.08.004
11. Havelek, R.; Muthna, D.; Tomsik, P.; Kralovec, K.; Seifrtova, M.; Cahlikova, L.; Hostalkova, A.; Safratova, M.; Perwein, M.; Cermakova, E.; et al. Anticancer potential of Amaryllidaceae alkaloids evaluated by screening with a panel of human cells, real-time cellular analysis and Ehrlich tumor-bearing mice. *Chem Biol Interact* **2017**, *275*. 121–132. https://doi.org/10.1016/j.cbi.2017.07.018
12. Lianza, M.; Verdan, M.H.; de Andrade, J.P.; Poli, F.; de Almeida, L.C.; Costa-Lotufo, L.V.; Neto, Á.C.; Oliveira, S.C.C.; Bastida, J. Batista, A.N.L.; et al. Isolation, Absolute Configuration and Cytotoxic Activities of Alkaloids from *Hippeastrum goianum* (Ravenna) Meerow (Amaryllidaceae). *J Braz Chem Soc* **2020**, *31*, 2135–2145. https://doi.org/10.21577/0103-5053.20200116
13. Sancha, S.A.R.; Gomes, A.V.; Loureiro, J.B.; Saraiva, L.; Ferreira, M.J.U. Amaryllidaceae-Type Alkaloids from *Pancratium maritimum*: Apoptosis-Inducing Effect and Cell Cycle Arrest on Triple-Negative Breast Cancer Cells. *Molecules* **2022**, *27*, 5759. https://doi.org/10.3390/molecules27185759
14. Trujillo, L.; Bedoya, J.; Cortés, N.; Osorio, E.H.; Gallego, J.-C.; Leiva, H.; Castro, D.; Osorio, E. Cytotoxic Activity of Amaryllidaceae Plants against Cancer Cells: Biotechnological, In Vitro, and In Silico Approaches. *Molecules* **2023**, *28*, 2601. https://doi.org/10.3390/molecules28062601
15. Zupkó, I.; Réthy, B.; Hohmann, J.; Molnár, J.; Ocsovszki, I.; Falkay, G. Antitumor activity of alkaloids derived from amaryllidaceae species. *In Vivo* **2009**, *23*, 41–48.
16. Evidente, A.; Kireev, A.S.; Jenkins, A.R.; Romero, A.E.; Steelant, W.F.A.; Van Slambrouck, S.; Kornienko, A. Biological Evaluation of Structurally Diverse Amaryllidaceae Alkaloids and their Synthetic

Derivatives: Discovery of Novel Leads for Anticancer Drug Design. *Planta Med* **2009**, *75*, 501–507. https://doi.org/10.1055/s-0029-1185340
17. Carvalho, K.R.; Silva, A.B.; Torres, M.C.M.; Pinto, F.C.L.; Guimarães, L.A.; Rocha, D.D.; Silveira, E.R.; Costa-Lotufo, L.V.; Braz-Filho, R.; Pessoa, O.D.L. Cytotoxic Alkaloids from *Hippeastrum solandriflorum* Lindl. *J Braz Chem Soc* **2015**, *26*, 1976–1980. https://doi.org/10.5935/0103-5053.20150176
18. Masi, M.; Van Slambrouck, S.; Gunawardana, S.; van Rensburg, M.J.; James, P.C.; Mochel, J.G.; Heliso, P.S.; Albalawi, A.S.; Cimmino, A.; van Otterlo, W.A.L.; et al. Alkaloids isolated from *Haemanthus humilis* Jacq., an indigenous South African Amaryllidaceae: Anticancer activity of coccinine and montanine. *S Afr J Bot* **2019**, *126*, 277-281. https://doi.org/10.1016/j.sajb.2019.01.036
19. Le, N.-T.-H.; De Jonghe, S.; Erven, K.; Vermeyen, T.; Baldé, A.M.; Herrebout, W.A.; Neyts, J.; Pannecouque, C.; Pieters, L.; Tuenter, E. Anti-SARS-CoV-2 Activity and Cytotoxicity of Amaryllidaceae Alkaloids from *Hymenocallis littoralis*. *Molecules* **2023**, *28*, 3222. https://doi.org/10.3390/molecules28073222
20. Le, N.-T.-H.; De Jonghe, S.; Erven, K.; Neyts, J.; Pannecouque C.; Vermeyen, T.; Herrebout, W.A.; Pieters, L.; Tuenter, E. A new alkaloid from *Pancratium maritimum* - Structure elucidation using computer-assisted structure elucidation (CASE) and evaluation of cytotoxicity and anti-SARS-CoV-2 activity. *Phytochem Lett* **2023**, *58*, 1–7. https://doi.org/10.1016/j.phytol.2023.09.006
21. Al Shammari, L.; Al Mamun, A.; Koutová, D.; Majorošová, M.; Hulcová, D.; Šafratová, M.; Breiterová, K.; Maříková, J.; Havelek, R.; Cahlíková, L. Alkaloid Profiling of *Hippeastrum* Cultivars by GC-MS, Isolation of Amaryllidaceae Alkaloids and Evaluation of Their Cytotoxicity. *Rec Nat Prod* **2020**, *14*, 154–159. https://doi.org/10.25135/rnp.147.19.06.1302
22. Rakhi, S.A.; Hara, Y.; Islam, Md.S.; Manome, T.; Alam, S.; Emon, N.U.; Al-Mansur, M.A.; Kuddus, Md.R.; Sarkar, Md.R.; Ishibashi, M.; et al. Isolation of bioactive phytochemicals from *Crinum asiaticum* L. along with their cytotoxic and TRAIL-resistance abrogating prospect assessment. *Heliyon* **2024**, *10*, e25049. https://doi.org/10.1016/j.heliyon.2024.e25049
23. Nair, J.J.; Rárová, L.; Strnad, M.; Bastida, J.; Cheesman, L.; van Staden, J. Crinane Alkaloids of the Amaryllidaceae with Cytotoxic Effects in Human Cervical Adenocarcinoma (HeLa) Cells. *Nat Prod Commun* **2014**, *9*, 461–466. https://doi.org/10.1177/1934578X1400900406
24. Silva, A.F.S.; de Andrade, J.P.; Machado, K.R.B.; Rocha, A.B.; Apel, M.A.; Sobral, M.E.G.; Henriques, A.T.; Zuanazzi, J.A.S. Screening for cytotoxic activity of extracts and isolated alkaloids from bulbs of *Hippeastrum vittatum*. *Phytomedicine* **2008**, *15*, 882–885. https://doi.org/10.1016/j.phymed.2007.12.001
25. Breiterová, K.; Koutová, D.; Maříková, J.; Havelek, R.; Kuneš, J.; Majorošová, M.; Opletal, L.; Hošťálková, A.; Jenčo, J.; Řezáčová, M.; et al. Amaryllidaceae Alkaloids of Different Structural Types from *Narcissus* L. cv. Professor Einstein and Their Cytotoxic Activity. *Plants* **2020**, *9*, 137. https://doi.org/10.3390/plants9020137
